# Is Whole-Body Cryostimulation Useful in Modulating Spasticity in Adults with Cerebral Palsy? A Case Study

**DOI:** 10.3390/jcm13247674

**Published:** 2024-12-16

**Authors:** Paolo Piterà, Matteo Bigoni, Elisa Prina, Boris Barrera, Duru Ceren Yavuz, Federica Verme, Jacopo Maria Fontana, Lorenzo Priano, Alessandro Mauro, Paolo Capodaglio

**Affiliations:** 1 Department of Neurosciences “Rita Levi Montalcini”, University of Turin, 10126 Turin, Italy; p.pitera@auxologico.it (P.P.); e.prina@auxologico.it (E.P.); lorenzo.priano@unito.it (L.P.); alessandro.mauro@unito.it (A.M.); 2 Laboratory of Clinical Neurobiology, IRCCS Istituto Auxologico Italiano, San Giuseppe Hospital, 28824 Verbania, Italy; 3 Division of Neurology and Neurorehabilitation, IRCCS Istituto Auxologico Italiano, 28824 Verbania, Italy; borisbarrera1994@gmail.com; 4 Faculty of Physical Therapy and Rehabilitation, Hacettepe University, 06100 Ankara, Turkey; durucerenyavuz@gmail.com; 5 Research Laboratory in Biomechanics, Rehabilitation and Ergonomics, IRCCS Istituto Auxologico Italiano, San Giuseppe Hospital, 28824 Verbania, Italy; f.verme@auxologico.it (F.V.); j.fontana@auxologico.it (J.M.F.); 6 Unit of Musculoskeletal and Metabolic Rehabilitation, IRCCS Istituto Auxologico Italiano, 20095 Milan, Italy; p.capodaglio@auxologico.it; 7 Department of Biomedical, Surgical and Dental Sciences, University “La Statale” of Milan, 20122 Milan, Italy

**Keywords:** cerebral palsy, spasticity, whole-body cryostimulation, rehabilitation

## Abstract

**Background**: This case study investigates the effect of a five-session whole-body cryostimulation (WBC) cycle on a 55-year-old female patient with cerebral palsy (CP) and lower limb spasticity (LLS) with a typical diplegic gait pattern. CP is a common physical disability characterized by motor impairments, including spasticity, which significantly impacts mobility and quality of life. The current treatments for spasticity often have limited efficacy and considerable side effects, making alternative therapies like WBC an area of interest. **Methods**: The patient underwent a 10-day inpatient rehabilitation program integrated with five WBC sessions at −110 °C for 2 min. The treatment effects were assessed immediately before and after the five WBC sessions using the Ashworth Scale, Fugl-Meyer Assessment, H-reflex test, and gait analysis. Psychosocial outcomes were measured with the SF-36, WHO-5, PSQI, ESS, and BDI questionnaires. **Results:** Immediately after the WBC cycle, gait analysis showed increased walking speed (0.48 to 0.61 m/s left; 0.49 to 0.57 m/s right) and step length (0.30 to 0.38 m left; 0.30 to 0.35 m right). The H/M ratio in the H-reflex test improved, indicating a better neuromuscular efficiency. Psychosocial assessments revealed a 42.5% reduction in pain and a 24% improvement in overall quality of life and well-being. **Discussion and Conclusions**: The objective improvements in gait parameters and neuromuscular modulation, along with the subjectively reported enhancements in functional abilities, highlight the potential of WBC as a valuable addition to rehabilitation strategies for this population. Further research is needed to confirm these findings and assess long-term outcomes.

## 1. Introduction

The Centers for Disease Control and Prevention define cerebral palsy (CP) as a collection of disorders that impair movement, posture, and balance [1] in affected individuals. CP represents the most prevalent physical disability in childhood, occurring in approximately two to three out of 1000 live births [2].

CP etiology is multifactorial, with various regions of the brain potentially affected, leading to a wide spectrum of clinical manifestations. Identified risk factors include preterm birth [3], perinatal infection such as chorioamnionitis [4], intrauterine growth restriction (IUGR) [5], administration of preterm antibiotics before membrane rupture, acidosis or asphyxia, and multiple gestation. Each of these factors can affect different regions of the brain and contribute to brain injury and CP development [2].

The clinical manifestations of CP are varied and encompass a broad range of abnormalities. Although permanent and nonprogressive, they can evolve over time. These disorders predominantly affect movement but also encompass other abnormalities, including impaired balance, reduced walking speed, and sensory deficits [6]. From a clinical perspective, reduced walking speed and the inability to ambulate safely in the community are significant ongoing health concerns and are associated with accelerated health decline as well as other adverse outcomes, including mortality, institutionalization, disability, and falls [7]. Several additional conditions often accompany CP, even if they are not included in its primary definition. These conditions commonly include pain (75%), intellectual disabilities (50%), difficulty walking (33%), hip dislocation (33%), speech impairment (25%), epilepsy (25%), incontinence (25%), and behavioral or sleep issues (20% to 25%) [2,8].

Furthermore, cognitive impairments are common in individuals with CP, often including learning disabilities, attention deficits, memory issues, and executive function difficulties [9,10,11].

Spastic CP is the most common type, representing about 70–80% of all diagnosed cases of such disease [12].

Spasticity is characterized by an abnormal increase in muscle tone and exaggerated reflexes, which significantly impair motor function, restrict mobility, and adversely affect the quality of life of individuals with CP [13]. In CP, spasticity can be combined with diplegia, hemiplegia, or quadriplegia, depending on the affected limbs [14]. Patients with lower limb spasticity (LLS) frequently exhibit secondary complications, including static or dynamic postural instability, limb deformity, contractures, and pain [15], and are also at higher risk of falls and inactivity [16].

With the increasing survival of preterm infants and increasing survival into adulthood, the overall prevalence in society continues to increase [17]. In the transition to adulthood, CP patients face many challenges, including a faster functional decline with age.

The diagnosis of cerebral palsy is primarily clinical and relies on recognizing its characteristic features [18]. It can be further categorized based on the type of movement disorder: spasticity (stiff muscles), dyskinesia (involuntary movements), ataxia (impaired coordination), or a combination of these types [18].

The current approach to managing spasticity is focused on physical rehabilitation, often supported by pharmacological treatments, to assist patients in achieving their goals. Evidence-based guidelines identify botulinum toxin type A (BoNT-A) injections as an effective first-line option for focal LLS [19]. Short-acting medications such as baclofen (Lioresal) and diazepam (Valium) are effective in relaxing muscle groups but are associated with numerous adverse effects, including sedation, dizziness, confusion, nausea, reduced seizure threshold, and central nervous system depression [20]. While these drugs may be required in the most severe cases (typically Gross Motor Function Classification System (GMFCS) levels IV or V), limited evidence supports their long-term use due to the risk of these side effects [21].

In addition to pharmacological options, advanced interventions for spasticity management in CP include intrathecal Baclofen therapy, which effectively reduces spasticity by delivering medication directly to the central nervous system, minimizing systemic side effects, and selective dorsal rhizotomy (SDR), which provides long-term reduction of spasticity and improvement in quality of life for selected patients [22]. However, intrathecal baclofen therapy carries risks such as infections, catheter dislocation, and pump malfunctions, while SDR, as a highly invasive procedure, may lead to transient paresthesias, muscle weakness, or other complications [22,23].

Additional treatment options include physiotherapy, orthosis, electrical stimulation of antagonist musculature, or localized cryotherapy. 

The local application of cold therapy promotes various effects, including post-injury reduction of inflammation, swelling, edema, muscle spasm, and pain and an increase in local circulation and joint range of motion [24,25]. A time-related reduction in spasticity has also been reported. The combination of cold therapy with physical and occupational therapy has been shown to reduce spasticity in children with spastic CP, translating into functional gains in hand function [26]. It could therefore be hypothesized that systemic cryotherapy may provide more generalized beneficial effects on spasticity and functioning. 

Whole-body cryostimulation (WBC) is a medical physical treatment that involves the whole body being exposed to extremely low temperatures (−110/−140 °C) for 2–3 min. Several studies have shown favorable outcomes for patients with various diagnoses affected by symptoms like chronic pain, fatigue, inflammation, and diffuse myalgia [27,28,29,30]. Moreover, WBC has shown clear efficacy in reducing spasticity in neurological conditions such as multiple sclerosis (MS) [31,32,33,34,35], significantly improving MS patients’ functional status and quality of life.

Studies have indicated that WBC can significantly decrease spasticity levels in MS patients, improving their overall functional status and quality of life. Additionally, research has demonstrated that WBC may be beneficial in post-stroke recovery by enhancing motor function and reducing muscle spasticity, thus promoting better rehabilitation outcomes. Previous research has demonstrated a reduction in spasticity severity following cryotherapy application in post-stroke patients [36,37]. In particular, the study by Krukowska et al., involving 56 patients with post-stroke spasticity, demonstrated that combining local cryotherapy with kinesitherapy was more effective in reducing spasticity rather than kinesiotherapy alone [36]. Similarly, Alcantara et al. found that cryotherapy applied to the calf muscles of individuals with chronic hemiparesis decreased muscle hypertonia, although it did not enhance the strength of dorsi- and plantar flexors or improve gait parameters [37]. Limited evidence also suggests the efficacy of local cryotherapy in improving spasticity in the muscles of the mouth [38], upper limbs, and hands [26] of children with CP.

No studies so far have investigated the effects of WBC on LLS and gait in adult patients with CP. Given that current pharmacological treatments for spasticity in CP patients present adverse effects, non-pharmacological complementary options appear appealing. Once contraindications have been excluded [39], WBC may represent a safe, non-invasive, and well-tolerated option to improve motor function and quality of life in this population.

Thus, the rationale for the selection of WBC in the management of CP spasticity is based on the initial evidence available on the effectiveness of this novel approach as a non-pharmacological and non-invasive intervention in modulating neuromuscular activity and reducing spasticity in various conditions through mechanisms such as decreased nerve conduction velocity, reduction of muscle spindle sensitivity, and inhibition of the segmental sensorimotor complex.

This case study aims to evaluate the impact of a five-session WBC cycle as an adjunctive intervention to a conventional physiotherapy-based gait training program on an adult CP patient with lower limb spasticity.

## 2. Materials and Methods

### 2.1. Case Description and Clinical History

As this case report was designed to explore the effects of whole-body cryostimulation (WBC) on spasticity in an individual with cerebral palsy (CP), no additional inclusion criteria were applied beyond the diagnosis of CP, reflecting the exploratory nature of the study. The exclusion criteria were limited to the contraindications specific to WBC [40], and each criterion was thoroughly assessed to ensure the patient’s eligibility for the intervention, as described in Section 2.3.2.

A 55-year-old Caucasian female CP patient was admitted to the Neurorehabilitation Unit of Piancavallo Hospital to undergo a 10-day rehabilitation program for optimizing gait and daily activities. She was born prematurely at seven months in a twin birth, with placenta previa. The patient presented with sequelae of cerebral palsy, characterized especially by residual typical diplegic spastic gait pattern and growth delay. Aside from motor deficits and spasticity, the patient did not present any cognitive impairments or psychiatric disorders associated with CP, either currently or documented in her medical history.

From ages 7 to 11, the patient underwent growth hormone therapy at our institute, achieving modest improvements in functional autonomy. Subsequently, no hormonal imbalances were detected. The patient also participated in psychomotor and neuromotor rehabilitation until reaching adulthood. She complained of severe generalized muscle spasticity, predominantly in the lower limbs and particularly in the left leg, and a pronated equinovalgus left foot.

In 2011, a treatment cycle with Botulinum toxin-A (BoNT-A) was initiated, targeting the calf muscles of the left leg, administered over a six-month period and later discontinued. Despite this treatment, the patient did not exhibit significant improvements and reported frequent falls towards the end of each cycle. Consequently, in 2012, the patient underwent functional surgery involving lengthening of the triceps surae muscle (according to the Vulpius procedure) for the left foot, followed by an inpatient rehabilitation program. This intervention led to improvements in balance and walking stability, with a noticeable reduction in falls, contributing to her current functional status and enhancing her ability to perform daily activities. She continued weekly rehabilitation sessions until the SARS-CoV-2 pandemic, which led to a period of inactivity and subsequent functional decline. Regular maintenance activities resumed only in the past year. For the past two years, the patient has reported an exacerbation of spasticity in the lower limbs, increased difficulty in ambulation, and greater fatigue sense, resulting in decreased autonomy in activities of daily living. During this period, she also experienced weight gain (from 43 to 50 kg; currently 47 kg at 148 cm), likely due to menopause. Additionally, the patient presented a history of chronic lower back pain, which improved following the left lower limb surgery. Lumbar MRI was performed in October 2023, revealing disk protrusions at L4–S1, spondyloarthritis, and features suggestive of possible sacroiliitis; a subsequent electromyographic study revealed mild lumbosacral radiculopathy at L4–L5 on both sides and, respectively, mild-to-moderate and moderate radiculopathy at S1 on the right and left sides. Diffuse tonic muscle activity was also found as an index of spastic dystonia in both lower limbs.

At the time of the study, the patient was not undergoing any pharmacological treatment for spasticity or other conditions.

### 2.2. Symptoms Assessment

To perform a clinical evaluation addressing spasticity, in particular, the Ashworth Scale (AS) was administered before the first cryostimulation treatment (pre-T0) and after the conclusion of the five-session cycle (post-T5). The Ashworth Scale (AS) was chosen because it is a widely recognized and validated clinical tool for assessing spasticity. This scale provides a standardized and simple way to evaluate changes in muscle tone before and after the intervention. Its clinical utility and ease of administration make it an ideal choice for tracking spasticity improvements over time.

Concurrently, the Fugl-Meyer Assessment for Lower Extremities (FMA-LE) was used to assess motor impairments in the lower limbs. The overall pain level was assessed using the Numeric Rating Scale (NRS) for pain. FMA-LE was chosen because it is a validated and widely used tool for evaluating motor impairments in individuals with neurological conditions. It assesses key motor functions, including reflexes, joint motion, and motor coordination, which align with the study’s objective of assessing the impact of the intervention on lower limb function.

In order to obtain objective and highly sensitive measurements of potential changes due to the introduction of the WBC cycle in the rehabilitation program, electromyography of the lower limbs and gait analysis were performed both before and after the WBC cycle.

The H-reflex test (electromyography) was conducted on the right (R SPI—Soleus) and left (L SPI—Soleus) Soleus nerves. The tibial nerve was stimulated with a square wave electrical pulse of 1 ms duration. The intensity of the stimulation gradually increased until maximal H-reflex (Hmax) and maximal M-response (Mmax) were obtained (45 mA). Recording electrodes were placed over the muscle belly of the Soleus muscle while stimulating electrodes were positioned on the skin overlaying the tibial nerve in the popliteal fossa. The latency (ms) (interval from the stimulus artifact to the onset of the H-reflex or M-response) and amplitude of the H-reflex and M-response (peak-to-peak) were recorded using a Synergy electromyograph (EMG) system.

The H-reflex test was included to obtain objective, quantitative, and highly sensitive measurements of neuromuscular function. This test specifically evaluates the reflex arc, including both sensory and motor pathways and measuring parameters such as latency and amplitude of the reflex responses (H and M), which provides detailed insights into the physiological mechanisms underlying potential changes in neuromuscular function.

Gait analysis was conducted both before and after the WBC cycle. Spatio–temporal and kinematic gait parameters were recorded with a six-camera motion-capture system (VICON, Oxford Metrics Ltd., Oxford, UK) operating at a sampling rate of 100 Hz, along with two force platforms (Kistler, Winterthur, Switzerland). A total of 22 spherical retro-reflective passive markers were placed at specific anatomical landmarks on the patient’s skin. Once this setup phase was completed, the patient was instructed to walk 10 m at a self-selected, natural pace. The raw 3D markers’ trajectories were then processed using the dedicated software (Polygon Application, version 2.4, VICON, Oxford Metrics Ltd., Oxford, UK). The spatio–temporal parameters of gait assessed included cadence, step time, step length, stride time, stride length, walking speed, double support time, single support time, foot off timing, opposite foot contact timing, and step width. The kinematic parameters assessed included: ankle dorsi/plantarflexion, knee and hip flexion/extension, and pelvic tilt. This method allows for the precise and objective evaluation of mobility and walking efficiency.

As the patient in this case did not exhibit significant cognitive issues, either currently or as documented in her medical history, no specific cognitive screening was performed as part of the study.

We assessed health-related quality of life using the Short Form Health Survey 36 (SF-36). Sleep quality was measured with the Pittsburgh Sleep Quality Index (PSQI), and the general level of daytime sleepiness was evaluated using the Epworth Sleepiness Scale (ESS). To assess overall subjective well-being, we utilized the five-item World Health Organization Well-Being Index (WHO-5). The severity of depression was assessed with the Beck Depression Inventory (BDI), while state and trait anxiety were measured using the State–Trait Anxiety Inventory (STAI), specifically STAI-1 for state anxiety and STAI-2 for trait anxiety. Psychosocial measures, including the SF-36, WHO-5, PSQI, ESS, BDI, and STAI, were selected for their validated reliability and relevance in evaluating the intervention’s impact on multidimensional quality of life, subjective well-being, and sleep quality, which are critical components of overall psychosocial health.

### 2.3. Intervention

#### 2.3.1. Physiotherapy and Adapted Physical Activity

The patient underwent a 1 h/day physiotherapy and adapted physical activity program that included a combination of cardiorespiratory endurance training and muscle strengthening exercises: continuous and rhythmic aerobic activities targeting major muscle groups (e.g., cycling, arm ergometry) and resistance exercises involving both multi-joint movements, such as lateral step-ups and squats, and single-joint movements, such as knee extensions. The program also incorporated passive and active/assisted mobilization of the upper and lower limbs, as well as the thoracic and lumbar spine (proprioceptive neuromuscular facilitation, PNF). Specific exercises for selective trunk control (core training), muscle strengthening, balance exercises, and coordination training were also included. Additionally, the patient was trained in using trekking poles to support gait training. Neurodynamic techniques were also part of the rehabilitation protocol. The program consisted of one-hour treatment sessions from Monday to Friday, complemented by 30 min cycling sessions with the same frequency. Passive stretching of spastic muscles, task-specific training, and education on reducing sedentary time and integrating functional exercises into daily activities were also provided.

#### 2.3.2. Whole-Body Cryostimulation (WBC)

**Figure 1:**
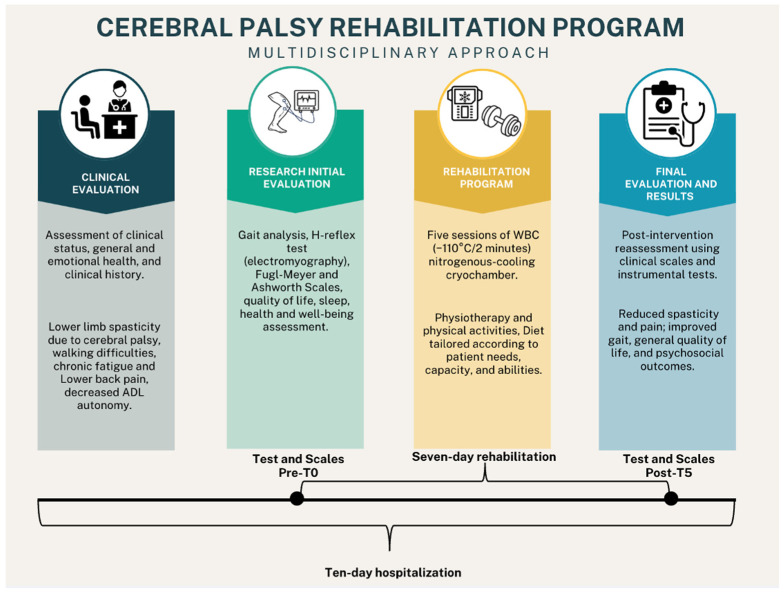
Graphical abstract of the study depicting rehabilitation intervention, tests and scales used for evaluation, and main results.

WBC consisted of five sessions, performed daily at 12 pm from 18 July 2024 to 24 July 2024. Contraindications to WBC were ruled out using Bad Voslau’s list of contraindications [40], which includes severe cardiovascular conditions, respiratory disorders, peripheral vascular diseases, acute infections, pregnancy, and severe psychiatric or cognitive impairments. The patient first underwent a 1 min familiarization session at −110 °C in a nitrogen-cooled cryochamber (Arctic, CryoScience, Rome, Italy), dressing in accordance with the rules applicable during WBC procedures, which included minimal clothing and protection for the body’s extremities. Subsequently, the patient underwent five consecutive WBC sessions, each lasting 2 min at −110 °C. All sessions were performed under the supervision of personnel specifically trained in WBC procedures. A schematic representation of the intervention is provided in the graphical abstract, Figure 1.

## 3. Results

Following five WBC sessions, improvements were noted across various domains, including physical functioning, emotional well-being, and sleep quality, suggesting a potential benefit of WBC in enhancing the overall quality of life for individuals with cerebral palsy. Detailed outcomes are presented in Table 1.

Significant reductions in pain and spasticity, alongside improvements in functional capacity, were observed following WBC sessions. These changes, evaluated through targeted assessments, are summarized in Table 2.

After the WBC cycle, the number of maximum M and H responses in the right Soleus increased, H latency decreased, and H amplitude significantly increased, indicating enhanced reflex activation and response speed. The H-reflex is an electrically evoked reflex assessing the integrity of the sensory and motor pathways, while the M-response represents the direct muscle response to electrical stimulation of the motor nerve.

The H/M ratio, which was pathological at baseline (indicating reduced neuromuscular transmission efficiency), improved markedly, suggesting improved neuromuscular efficiency. Additionally, maximum M and H amplitudes increased, and M latency decreased, indicating overall improved neuromuscular response speed. Conversely, the left Soleus (L SPI—Soleus) showed a decrease in reflex responses and a slight increase in H latency but a minor improvement in H amplitude and H/M ratio, with decreased M latency indicating some neuromuscular improvement. All data regarding the H-reflex test are reported in Table 3. 

The results of the gait analysis indicate significant improvements in several parameters after the WBC treatment. Cadence increased slightly, indicating improved walking rhythm and stability. Walking speed improved from 0.48 m/s to 0.61 m/s on the left and from 0.49 m/s to 0.57 m/s on the right, suggesting enhanced mobility and endurance. Step and stride lengths increased, reflecting better stride efficiency and a more extended gait pattern. Double support time remained nearly unchanged, while a slight bilateral improvement in single support time was detected, suggesting better balance and control. Data of gait analysis pre- and post-WBC are reported in Table 4.

**Figure 2:**
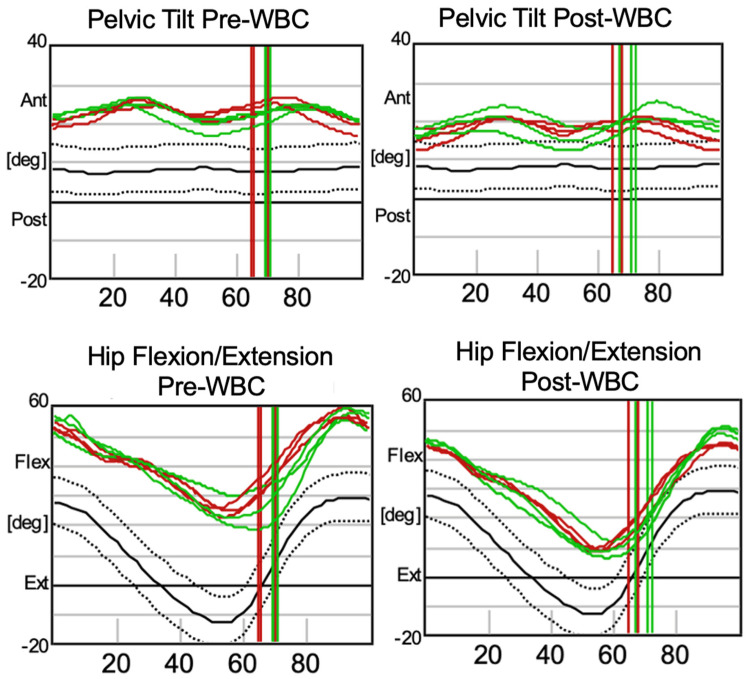
Kinematic analysis of pelvic tilt and hip flexion/extension before (pre-WBC) and after (post-WBC) the WBC cycle.

**Figure 3:**
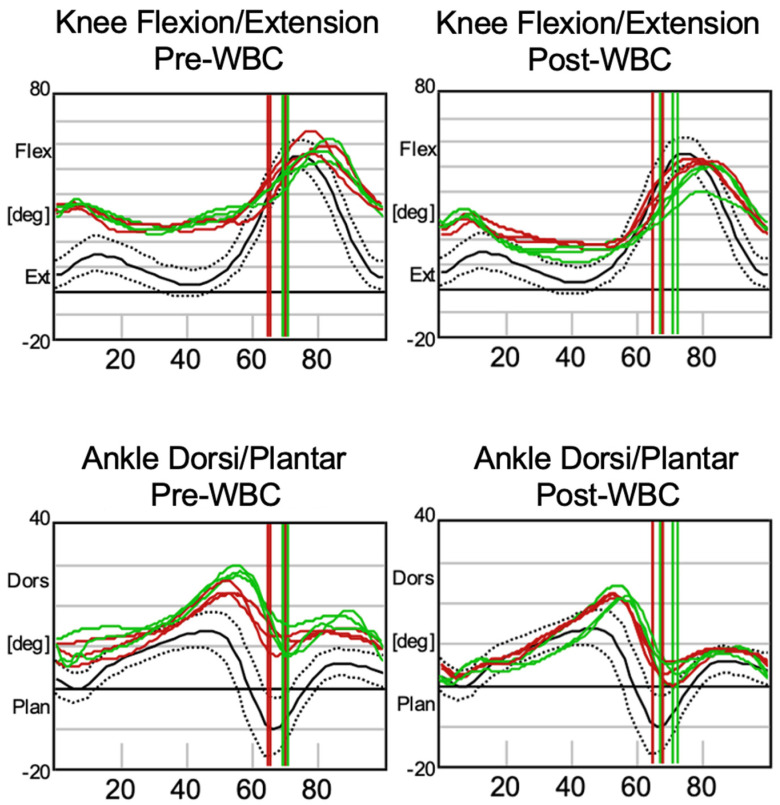
Kinematic analysis of knee flexion/extension and ankle dorsiflexion/plantarflexion before (pre-WBC) and after (post-WBC) the WBC cycle.

Other noticeable changes are evident in the kinematic data presented in Figure 2 and Figure 3, which can be interpreted with the following reading key: the right marker trajectory is represented by the horizontal green line, the left marker trajectory by the horizontal red line, the normative range of motion by the area within the two dotted lines, and the mean normal trajectory by the black line. The right and left foot-off points during the gait cycle are indicated by the green and red vertical lines, respectively.

In particular, as shown in Figure 2, the first row of graphs illustrates pelvic tilt before and after treatment. A reduced degree of anterior tilt (antiflexion) is noticeable post-treatment, although the characteristic double-bump typical of gait in CP patients remains present. Moreover, for hip flexion/extension, described in the second row of graphs, an after-treatment shift toward a better hip extension is evident, nearly reaching the physiological range of motion. These findings suggest enhanced trunk and lower limb control, as well as a more physiological gait pattern during both the swing and stance phases of the gait cycle.

Figure 3 provides the kinematic data for the lower joints of the legs. The first row of graphs highlights knee flexion/extension, where the post-treatment results indicate improved physiological extension during the stance phase and at the end of the swing phase. Similarly, ankle dorsiflexion/plantarflexion, depicted in the second row of graphs, shows increased plantarflexion, particularly during the swing phase. These findings collectively suggest better muscle activation and reduced spasticity.

## 4. Discussion

This case report aimed at providing initial evidence of the impact of a five-session WBC cycle implemented into a traditional rehabilitation program on severe LLS in an adult patient with CP. The patient underwent a multi-component rehabilitation program with multiple interventions (cardiorespiratory endurance training, resistance exercises involving both multi-joint movements, passive and active/assisted mobilization of the upper and lower limbs, balance exercises, coordination training, gait training, neurodynamic techniques), and, to minimize the impact of all other interventions rather than WBC, the outcomes were measured immediately before and after the WBC cycle. The findings from this case study suggest that WBC is a well-tolerated, non-invasive, complementary intervention for modulating spasticity and improving overall functional outcomes, including gait parameters and psychosocial well-being. Spasticity, characterized by a velocity-dependent increase in resistance to stretch, is commonly associated with exaggerated stretch reflexes. The application of systemic cold therapy has been shown to temporarily alleviate spastic conditions by decreasing resistance to passive movement, reducing hyperactive stretch reflexes, and minimizing clonus. However, whether cold therapies might have adverse effects on muscle mechanical properties is still debated. Some studies seem to suggest that local air-pulsed cryotherapy can induce an increase in muscle stiffness that may lower the amount of stretch that the muscle tissue is able to sustain without subsequent injury [41,42,43,44]. Possible underlying mechanisms that explain how cold therapy could reduce spasticity include the ones described by Eldred et al. [45], Ottoson [46], and Knutsson and Mattsson [47], suggesting that cold reduces muscle tone by decreasing spindle sensitivity. This reduction is due to a temperature-dependent decrease in the rate of spontaneous spindle discharge and Golgi tendon organ activity. Additionally, Michlovitz et al. [48] suggested that cryotherapy might inhibit the segmental sensorimotor complex, affecting large afferent fibers, muscle spindles, skin receptors, extrafusal muscle fibers, and the myoneural junction. Another explanation, proposed by Lippold et al. [49], involves changes in membrane polarization, where cooling induces hyperpolarization, thus reducing or abolishing spindle discharge. Miglietta [50] noted that significant muscle temperature reduction is necessary to abolish clonus and spasticity, possibly due to sympathetic fiber stimulation, which decreases spindle sensitivity through vasoconstriction. Finally, studies by Urbscheit et al. [51] support these findings, showing that deep, prolonged cold can induce muscle relaxation by lowering the background level of stretch afferent input and suggesting that cryotherapy can reduce spasticity and improve joint range of motion. Interestingly, Urbscheit investigated changes in H-response in hemiplegic patients after cold application and found that the patients responded differently, suggesting that local cooling might decrease, increase, or exert no effect on spasticity [51]. 

The results of our case study align with previous observations showing a beneficial effect on spasticity in several conditions, including CP [26], indicating that cold therapy can provide short-term relief from spasticity.

At the clinical level, the scale assessing spasticity indicated a notable reduction, as evidenced by a 50% decrease on the Ashworth Scale. The Fugl-Meyer Assessment showed moderate improvements following cryotherapy intervention, with increases in motor function (+8.8%), sensation (+16.6%), and passive joint movements (+20%). Additionally, there was a significant reduction in pain, as reflected by a 40% decrease in the NRS pain score. These results suggest that the intervention had an objective impact on pain management and functional improvements, though the overall improvements might be partially limited by the sensitivity of the scales used, particularly given the brief duration of the intervention.

At a subclinical level, neurophysiological and biomechanical instrumental tests (H-reflex test and 3D gait analysis) showed interesting findings. The H/M ratio decreased from a pathological to a normal level, suggesting an improvement in neuromuscular efficiency. Both H- and M-amplitudes increased significantly, potentially indicating enhanced reflex responsiveness, which might be due to improved muscle activation or reduced inhibitory mechanisms post-treatment. Furthermore, the reduction in both H and M latencies post-WBC indicates faster neuromuscular response times, which is a positive outcome suggesting enhanced neural conduction or synaptic efficiency. Interestingly, the most significant improvements in H-reflex parameters were observed in the right leg, which initially displayed more pathological values compared to the left leg, despite the left leg exhibiting higher levels of spasticity. A previous surgical intervention performed on the left leg in 2013 (lengthening of the triceps surae muscle) may have accounted for these findings. Such a procedure likely altered the neuromuscular architecture and reflex dynamics of the left leg, leading to more normalized H-reflex values even before the intervention with WBC. Therefore, it is plausible that the left leg had limited scope for further improvement subsequent to the WBC. 

Three-dimensional gait analysis revealed significant improvements in several spatio–temporal and kinematic parameters following the WBC cycle. Notably, there was an increase in cadence and walking speed, suggesting enhanced mobility and endurance, reflecting the patient’s ability to walk more efficiently as a result of spasticity decrease. Additionally, the increase in step and stride lengths indicates better stride efficiency and a more extended gait pattern, which may contribute to a smoother and more stable walking experience. Furthermore, the most significant gait findings concern the cinematic parameters of hip extension and knee and ankle flexion, which are correlated with changes in spasticity in accordance with the H-reflex changes.

In line with previous evidence from healthy and pathological subjects [27,52,53,54,55], the psychosocial assessments before and after the WBC cycle revealed significant improvements in the patient’s reported quality of life. The Short Form Health Survey 36 (SF-36) demonstrated substantial increases in physical functioning, emotional well-being, social functioning, and pain reduction. Notably, the pain score improved markedly, and the overall health score showed a positive change. The WHO-5 Well-Being Index also improved significantly, indicating enhanced subjective well-being. Additionally, sleep quality, as assessed by the Pittsburgh Sleep Quality Index (PSQI), improved, reflected by a reduction in sleep disturbance scores. The Epworth Sleepiness Scale (ESS) score decreased, suggesting a reduction in daytime sleepiness. Although the Beck Depression Inventory (BDI) score and the State–Trait Anxiety Inventory (STAI) scores showed only minimal improvements, it is important to note that the initial results were not within the pathological range. The overall trend suggests that WBC not only benefits physical function but also contributes positively to the patient’s overall quality of life. The reductions in pain and spasticity likely played a role in these positive outcomes, as improved physical comfort can lead to better mood and sleep patterns.

Because of the lifelong spasticity experienced by the patient, the results of this intervention appear particularly valuable.

While we acknowledge that the reported improvements cannot be definitively ascribed to WBC due to concurrent rehabilitation interventions, the patient described a subjective reduction in muscle stiffness that was not previously experienced with other rehabilitation treatments. This outcome is supported by specific and objective assessments, which confirmed reductions in spasticity and improvements in neuromuscular function. While the improvements observed align with prior studies, further research is necessary to determine whether these changes can be directly attributed to WBC or are influenced by concurrent interventions and placebo effects.

Potential confounders in this study include the concurrent physiotherapy-based rehabilitation program, which incorporated exercises targeting neuromuscular function, and the patient’s positive emotional response to the intervention, which could have amplified the placebo effect. To address these confounding factors, the study outcomes were measured immediately before and after the WBC cycle, minimizing the influence of other interventions performed during the rehabilitation program. Additionally, the consistency of the positive findings across multiple outcome measures, including subjective reports, clinical scales, and objective instrumental assessments, strengthens the validity of the observed improvements. However, the placebo effect, driven by the patient’s positive emotional response and satisfaction with a brief, well-tolerated intervention free from adverse effects, cannot be entirely ruled out.

This case study has several limitations. The single-subject design and the brief duration of the intervention restrict the generalizability of the findings and preclude long-term outcome assessment. Moreover, the absence of a control condition makes it challenging to isolate the specific contribution of WBC from other rehabilitation components. Despite these limitations, this study highlights the innovative potential of WBC as a complementary intervention for managing spasticity and improving motor and psychosocial outcomes in adults with CP.

Future studies should involve larger sample sizes, include control groups, and implement long-term follow-up to determine the sustained effects of WBC and its specific contribution to rehabilitation outcomes. Further exploration of WBC’s mechanisms of action, particularly its impact on neuromuscular function and spasticity, is also warranted to validate its role as an evidence-based option in CP rehabilitation.

This present case study is innovative because it demonstrates the potential benefits of WBC in spasticity in adults with CP. This finding supports expanding the scope of WBC application proposing new research in this area, thus widening the rehabilitative options for CP patients.

## 5. Conclusions

In conclusion, this case study provides initial anecdotal evidence that WBC can be an effective adjuvant intervention in addition to a traditional rehabilitation program for modulating spasticity and improving functional and psychosocial outcomes in the short-term in adults with cerebral palsy.

The objective improvements in gait parameters and neuromuscular modulation, along with the subjectively reported enhancements in functional abilities, highlight the potential of WBC as a valuable addition to rehabilitation strategies for this population.

Further research with larger sample sizes and longer follow-up periods is warranted to confirm these findings and explore the long-term benefits of WBC in the management of spasticity and its associated functional impairments.

## Figures and Tables

**Table 1:** Summary of psychosocial and functional outcomes before and after the WBC cycle.

Outcome and Range	Pre	Post	Δ%
SF-36			
*Physical functioning*	30%	65%	+35%
*Role limitations due to physical health*	100%	100%	0
*Role limitations due to emotional problems*	100%	100%	0
*Energy/fatigue*	45%	55%	+10%
*Emotional well-being*	64%	80%	+16%
*Social functioning*	25%	50%	+25%
*Pain*	45%	87.5%	+42.5%
*General health*	55%	60%	+5%
WHO-5 (0–100)	68	92	+24%
PSQI (0–21)	7	3	−19.04%
ESS (0–24)	6	2	−16.7%
BDI (0–63)	7	2	−7.9%
STAI 1 (20–80)	33	33	0
STAI 2 (20–80)	38	35	−3.8%

Abbreviation: SF-36: Short Form Health Survey (36 items); BDI: Beck Depression Inventory; WHO-5: World Health Organization Well-Being Index (0–100); PSQI: Pittsburgh Sleep Quality Index; ESS: Epworth Sleepiness Scale; STAI 1: State–Trait Anxiety Inventory (State); STAI 2: State–Trait Anxiety Inventory (Trait).

**Table 2:** Results of pain, spasticity, and motor impairment assessments.

Outcome and Range	Pre	Post	Δ%
NRS pain (0–10)	8	4	−40%
FUGL-MEYER			
*Motor Function (0–34)*	17	20	+8.8
*Sensation (0–12)*	8	10	+16.6%
*Passive Joint Movement (0–20)*	16	20	+20%
*Joint pain (0–20)*	0	4	+20%
ASHWORTH SCALE (0–4)	2	0	−50%

**Table 3:** H-reflex test (electromyography) assessed before and after the WBC cycle.

NERVE	Test	Resp. No M Max	Resp. No H Max	Lat. H (ms)	H Amp. (mV)	H/M Amp. (%)	M Amp. M Max (mV)	M Amp. H Max (mV)	Lat. M M Max (ms)	Lat. MH Max (ms)
R SPI-SOLEUS	T0	8	8	25.5	1.6	95.3	1.7	1.7	9.45	9.45
R SPI-SOLEUS	T5	10	10	24.25	3.7	56.2	6.6	6.6	2.95	2.95
L SPI-SOLEUS	T0	7	7	24.25	0.8	18.7	4.3	4.3	7.4	7.4
L SPI-SOLEUS	T5	1	1	26.7	0.9	30.3	3.1	3.1	3.5	3.5

Abbreviation: R SPI—Soleus: Right Soleus; L SPI—Soleus: Left Soleus, Resp. No M Max: Maximum number of M responses; Resp. No H Max: Maximum number of H responses; Lat. H: Latency of the H-reflex H Amp.: Amplitude of the H-reflex H/M Amp.: Ratio of H-reflex amplitude to M-response amplitude; M Amp. M Max (mV): Maximum amplitude of the M-response; M Amp. H Max (mV): Maximum amplitude of the H-reflex Lat. M M Max: Latency of the maximum M-response; Lat. M H Max: Latency of the maximum H-reflex.

**Table 4:** Gait analysis results before and after WBC cycle.

Parameter	Pre-WBC (Left)	Pre-WBC (Right)	Post-WBC (Left)	Post-WBC (Right)	Normal Range
Cadence (steps/min)	96.2 ± 9.72	36.2 ± 2.41	98.0 ± 1.99	37.3 ± 2.13	113 ± 12.4
Double Support (%)	35.1 ± 4.23	36.2 ± 2.41	37.7 ± 1.63	37.3 ± 2.13	23.6 ± 3.75
Foot Off (%)	66.7 ± 2.60	69.6 ± 0.62	66.6 ± 1.41	69.8 ± 2.31	61.6 ± 2.39
Opposite Foot Contact (%)	48.0 ± 2.12	51.5 ± 1.27	48.1 ± 0.46	50.7 ± 0.55	50.1 ± 2.24
Opposite Foot Off (%)	16.5 ± 4.17	18.1 ± 1.74	19.3 ± 1.44	18.2 ± 1.64	11.9 ± 2.16
Single Support (%)	31.5 ± 2.13	33.4 ± 2.98	28.8 ± 1.00	32.4 ± 1.15	38.2 ± 2.60
Step Length (m)	0.30 ± 0.058	0.30 ± 0.053	0.38 ± 0.024	0.35 ± 0.024	0.62 ± 0.081
Step Time (s)	0.66 ± 0.058	0.61 ± 0.060	0.64 ± 0.010	0.60 ± 0.010	0.49 ± 0.24
Step Width (m)	0.15 ± 0.012	0.14 ± 0.011	0.14 ± 0.0016	0.15 ± 0.015	0.11 ± 0.02
Stride Length (m)	0.60 ± 0.12	0.61 ± 0.11	0.75 ± 0.033	0.69 ± 0.033	1.22 ± 0.15
Stride Time (s)	1.27 ± 0.15	1.25 ± 0.098	1.23 ± 0.027	1.22 ± 0.034	1.06 ± 0.12
Walking Speed (m/s)	0.48 ± 0.11	0.49 ± 0.12	0.61 ± 0.023	0.57 ± 0.043	1.17 ± 0.23

## Data Availability

The data presented in this study are available within the article.
